# LDL-Dependent Regulation of TNFα/PGE_2_ Induced COX-2/mPGES-1 Expression in Human Macrophage Cell Lines

**DOI:** 10.1007/s10753-022-01778-y

**Published:** 2023-01-04

**Authors:** Frank Neuschäfer-Rube, Theresa Schön, Ines Kahnt, Gerhard Paul Püschel

**Affiliations:** 1 Institut Für Ernährungswissenschaft, Universität Potsdam, Arthur-Scheunert-Allee 114-116, 14558 Nuthetal, Germany; 2 Institut Für Ernährungswissenschaft, Universität Giessen, Wilhelmstr. 20, 35392 Gießen, Germany

**Keywords:** Prostaglandin E2, LDL, PI3K, Signal transduction

## Abstract

Inflammation is a hallmark in severe diseases such as atherosclerosis and non-alcohol-induced steatohepatitis (NASH). In the development of inflammation, prostaglandins, especially prostaglandin E_2_ (PGE_2_), are major players alongside with chemo- and cytokines, like tumor-necrosis-factor alpha (TNFα) and interleukin-1 beta (IL-1β). During inflammation, PGE_2_ synthesis can be increased by the transcriptional induction of the two key enzymes: cyclooxygenase 2 (COX-2), which converts arachidonic acid to PGH_2_, and microsomal prostaglandin E2 synthase 1 (mPGES-1), which synthesizes PGE_2_ from PGH_2_. Both COX-2 and mPGES-2 were induced by a dietary intervention where mice were fed a fatty acid-rich and, more importantly, cholesterol-rich diet, leading to the development of NASH. Since macrophages are the main source of PGE_2_ synthesis and cholesterol is predominantly transported as LDL, the regulation of COX-2 and mPGES-1 expression by native LDL was analyzed in human macrophage cell lines. THP-1 and U937 monocytes were differentiated into macrophages, through which TNFα and PGE-2 induced COX-2 and mPGES-1 expression by LDL could be analyzed on both mRNA and protein levels. In addition, the interaction of LDL- and EP receptor signal chains in COX-2/mPGES-1 expression and PGE_2_-synthesis were analyzed in more detail using EP receptor specific agonists. Furthermore, the LDL-mediated signal transduction in THP-1 macrophages was analyzed by measuring ERK and Akt phosphorylation as well as transcriptional regulation of transcription factor Egr-1. COX-2 and mPGES-1 were induced in both THP-1 and U937 macrophages by the combination of TNFα and PGE_2_. Surprisingly, LDL dose-dependently increased the expression of mPGES-1 but repressed the expression of COX-2 on mRNA and protein levels in both cell lines. The interaction of LDL and PGE_2_ signal chains in mPGES-1 induction as well as PGE_2_-synthesis could be mimicked by through simultaneous stimulation with EP2 and EP4 agonists. In THP-1 macrophages, LDL induced Akt-phosphorylation, which could be blocked by a PI3 kinase inhibitor. Alongside blocking Akt-phosphorylation, the PI3K inhibitor inhibited LDL-mediated mPGES-1 induction; however, it did not attenuate the repression of COX-2 expression. LDL repressed basal ERK phosphorylation and expression of downstream transcription factor Egr-1, which might lead to inhibition of COX-2 expression. These findings suggest that simultaneous stimulation with a combination of TNFα, PGE_2_, and native LDL-activated signal chains in macrophage cell lines leads to maximal mPGES-1 activity, as well repression of COX-2 expression, by activating PI3K as well as repression of ERK/Egr-1 signal chains.

**Supplementary Information:**

The online version contains supplementary material available at 10.1007/s10753-022-01778-y.

## INTRODUCTION

There is increasing evidence that inflammatory disorders are a hallmark of severe chronic diseases such as atherosclerosis, non-alcohol-induced steatohepatitis (NASH), inflammatory bowel diseases, and rheumatoid arthritis [1–3]. Inflammation is a complex process with a broad spectrum of mediators been involved such as cytokines, chemokines, and as major players, the prostaglandins [4]. Prostaglandins are formed when the C-20 unsaturated fatty acid arachidonic acid is released from the plasma membrane by phospholipases (PLA_2s_). Arachidonic acid is then metabolized by the sequential actions of prostaglandin G/H synthase (cyclooxygenase, COX) and the respective synthases. The cyclooxygenase exists as two distinct enzymes referred to as COX-1 and COX-2. Among these bioactive lipids, PGE_2_ is a pivotal prostaglandin that regulates multiple biological processes both under normal and pathological conditions [5]. In addition to its function as key mediator in inflammation, PGE_2_ plays a key role in physiologic cellular events such as female reproduction, regulation of vascular tension, kidney and neuronal functions, and gastric mucosal protection. In inflammation, PGE_2_ and its specific G protein-coupled EP receptors (EP1-EP4) are involved in the fine tuning of the inflammatory response [6].

Proteins involved in PGE_2_ synthesis and signalling have been shown to be increased in inflammation [7]. Thus, the expression of COX-2, mPGES-1, and PGE_2_ receptors (EP-R) was increased in inflammatory regions of atherosclerotic plaques of patients with carotid stenosis and the proteins were colocalized in the plaque cells [8]. Dietary lipids, especially cholesterol, are important factors in the progress of inflammation and PGE_2_ synthesis. In mice, a high fat/high cholesterol containing diet fat induced a “non-alcohol-induced-steato-hepatitis (NASH)” whereas a high fat diet without cholesterol did not [9–11]. In the liver of NASH-mice the number of infiltrating macrophages was increased whereas the expression of both COX-2 and mPGES-1 was induced [12]. Dietary cholesterol is transported to the liver from peripheral organs as chylomicron remnants, which are converted to VLDL, IDL, and finally to cholesterol-rich low-density-lipoprotein (LDL). After leaving the liver, LDL may serve as a source for cholesterol in any cell of the body. In addition, LDL can be oxidatively modified in vivo in oxidation events that include phospholipids, fatty acids, and Apo B modifications [13]. Oxidation of LDL can increase its proinflammatory and proatherogenic properties as oxLDL can be taken up by macrophages via scavenger receptors A and B (SR-A and SR-B (CD36) or lectin-like oxLDL receptor (LOX-1) leading to foam cell formation [14]. The knowledge regarding the regulation of COX-2 and mPGES-1 in macrophages by native or oxLDL is limited and sometimes controversial. In primary human macrophages, ox-LDL repressed LPS-induced COX-2 expression [15], whereas in THP-1 macrophages, oxLDL increased COX-2 expression [16]. In the U937 macrophage cell line, the cholesterol oxidation product 27-hydroxycholesterol induced both the expression of COX-2 and mPGES-1 [17]. Little to nothing is known about the regulation of COX-2/mPGES-1 expression by native LDL under inflammatory conditions.

The aim of the current study was to analyze the role of native LDL as a modulator of TNFα/PGE_2_-induced COX-2/mPGES-1 expression in human macrophage cell lines.

Our data indicates that native LDL enhanced the TNFα/PGE_2_-elicited induction of mPGES-1 in macrophages by an activation of PI3K whereas COX-2 expression was repressed, perhaps due to a reduction of ERK phosphorylation and subsequent repression of Egr-1 expression.

## MATERIALS AND METHODS

### Materials

All chemicals were purchased from commercial sources indicated throughout the text. Oligonucleotides were custom-synthesized by Biolegio (Nijmegen, Netherlands).

EP receptor specific agonists 19(R)-hydroxy prostaglandin E2 (EP2 agonist), CAY10598 (EP4 agonist), and the PGE_2_ ELISA were from Cayman chemicals (Ann Arbor, USA). Antibodies used were GAPDH (sc-2578) and mPGES-1 (sc-365844) from Santa Cruz Biotechnology (Heidelberg, Germany), COX-2 (12282), Akt (9272) and pAkt Ser-473 (4060), ERK (9102), pERK (9106), pIKK α/β Ser176/180 (2694), and cleaved caspase-3 Asp175 (9661) from Cell Signalling (Frankfurt, Germany). Human LDL was purchased from LEE Biosolutions (Maryland Heights, USA).

### Cell Culture and Treatment

THP-1 und U937 monocytes were cultured in very low endotoxin RPMI1640 containing 10% heat-inactivated FCS and antibiotics. Monocytes were seeded in 35 mm dishes (10^6^ cells/plate) in culture medium and differentiated to macrophages by the addition of 100 ng/ml PMA for 24 h. Cells were washed two times with serum-free medium and incubated with medium containing 0.5% FCS for another 24 h. Cells were then treated for 24 h, washed two times with PBS and then transferred to liquid nitrogen.

### Cell Viability Assay (Alamar Blu)

Cell viability was analyzed by measuring the conversion of non-fluorescent resazurin to fluorescent resorufin by viable THP-1 or U937 macrophages (Alamar Blue assay) [18]. Cells were plated in 96-well plates (10^5^ cells/well) and differentiated to macrophages as described above. After serum-starvation macrophages were stimulated with 50 ng/ml TNFα + 1 µM PGE_2_ and increasing LDL-concentrations for 24 h. Then, cells were washed and incubated with 0.1 mg/ml resazurin in RPMI 1640 for 2 h. Resorufin fluorescence was measured every 30 min (Em 544 nm;Exc 590 nm).

### MDA Assay

Differentiated THP-1 cells were incubated in culture medium (RPMI 16,040 + 0.5% (v/v) FCS) with or without 500 µg/ml native LDL for 0 h or 24 h at 37 °C. Then, malondialdehyde as a product of lipid peroxidation [19] was measured in cell culture supernatants using a commercial TBARS assay kit (Cayman chemicals, item 10009055) according to the manufacturer’s instructions.

### Real-Time RT-PCR

Cells were stimulated with 1 µM PGE_2_, 1 µM EP-R agonists and/or 50 ng/ml TNFα in the presence of native human LDL for the time indicated and washed with PBS. Total RNA was isolated from treated cells using Peqgold total RNA kit (Peqlab, Germany). One microgram total RNA were reverse transcribed into cDNA using oligo dT primers and an M-MuLV Reverse Transcriptase (Thermo Scientific, Germany). Hot start real- time PCR for the quantification of each transcript was carried using 2 × Maxima SybrGreen qPCR mix (Thermo Scientific), 0.25 µM of each primer and 2.5 µL of cDNA, which was diluted 1:10. PCR was performed with an initial enzyme activation step at 95 °C for 10 min, followed by 42 cycles of denaturation at 95 °C for 30 s, annealing at 57 °C for 30 s, and extension at 72 °C for 30 s in a real-time DNA thermal cycler (CFX96™, 10 µl reaction volume, BIO-RAD; Munich). The oligonucleotides used are listed in Table 1. The expression level was calculated as an n-fold induction of the gene of interest (int) in treated versus control cells with GAPDH, actin, or HPRT as reference genes (ref). The calculation is based on the differences in the threshold cycles between control (c) and treated (t) groups according to the formula: $\mathrm{fold \ induction}={2}^{\left(\mathrm{c}-\mathrm{t}\right)\mathrm{int}}/{2}^{\left(\mathrm{c}-\mathrm{t}\right)\mathrm{rev}}$. For the final N-fold calculation, the mean of n-fold calculations using different reference genes was determined. For the calculation of EP-R or COX-1/2 copy numbers of plasmids with cloned cDNAs coding for EP-R, COX-1/2 and GAPDH were used as templates to prepare standard curves with defined copy numbers.

**Table 1:** Oligonucleotide Primers Used for Real-Time qPCR

Gene	Forward	Reverse
GAPDH	5′-TGATGACATCAAGAAGGTGG	5′-TTACTCCTTGGAGGCCATGT
Actin	5′-CCCAGCCATGTACGTTGCTAT	5′-GGGTGGCTTTTAGGATGGCAA
HPRT	5′-AGGGACTGAACGTCTTGCTCG	5′-ATCCAACACTTCGTGGGGTC
COX-1	5′-CTCCGGTTCTTGCTGTTCCT	5′-GTCACACTGGTAGCGGTCAA
COX-2	5′-TGTGCCTGATGATTGCCCGA CTCC	5′-TGTTGTGTTCCCGCAGCCAGATTG
mPGES-1	5′-GAAGAAGGCCTTTGCCAACCC	5′-GTGCATCCAGGCGACAAAAG
EP1	5′-TCGCTTCGGCCTCCACCTTCTTTG	5′-CGTTGGGCCTCTGGTTGTGCTTAG
EP2	5′-CGAGACGCGACAGTGGCTTCC	5′-CGAGACGCGGCGCTGGTAGA
EP3	5′-CGGGGCTACGGAGGGGATGC	5′-ATGGCGCTGGCGATGAACAACGAG
EP4	5′-TCGCGCAAGGAGCAGAAGGAGACG	5′-GGACGGTGGCGAGAATGAGGAAGG

Accession numbers for the genes were as folles: GAPDH (AB062273), Actin (NM_001101), HPRT (NM_000194), COX-1 (NM_000962), COX-2 (NM_000963), mPGES-1 (NM_025072/NM_198938), EP1 (L22647), EP2 (NM_000956), EP3 (E15918), and EP4 (NM_000958).

### PGE_2_ ELISA

Cells were stimulated with 1 µM EP2 + EP4 agonists + 50 ng/ml TNFα in the absence or presence of 500 µg/ml native human LDL for 24 h. Then, supernatants were collected and processed for PGE_2_ quantification by competitive sandwich ELISA according to the manufacturer’s instructions.

### Western Blot Analysis

THP-1 and U937 cells were stimulated with 1 µM PGE_2_, 1 µM EP2/EP4-R agonist, and/or 50 ng/ml TNFα in the presence of native human LDL for the time indicated. Cells were lysed in lysis buffer (20 mM Tris pH 7.4, 150 mM NaCl, 1 mM ethylenediaminetetraacetic acid (EDTA), 1 mM ethylene glycol tetraacetic acid (EGTA), 1% (v/v) Triton X-100, 2.5 mM sodium pyrophosphate, 1 mM ß-glycerolphosphate, 50 mM NaF, protease inhibitors, and 1 mM sodium orthovanadate), homogenized by sonication and insoluble material was removed by centrifugation (10,000 × *g*, 15 min, 4 °C). Protein content was determined using Bradford assay [20]. Proteins were resolved by SDS-PAGE and transferred to a polyvinylidene difluoride (PVDF) membrane. Membranes were blocked in 5% non-fat dry milk in 20 mM Tris, 136 mM NaCl, and 0.1% (v/v) Tween (TBS/Tween) for 1 h at room temperature and incubated with the first antibody in TBS/Tween containing 5% bovine serum albumin overnight at 4 °C and a horseradish-peroxidase-conjugated anti-rabbit or anti-mouse IgG for 2 h at room temperature. Visualization of immune complexes was performed using a Clarity chemiluminescence reagent (BioRad, München).

### Statistical Analysis

To correct for differences in sensitivity of different cell charges towards the stimuli, values were normalized to average inducibility defined as the mean of values obtained for as indicated: control, TNFα + PGE_2_ and TNFα + PGE_2_ + 500 µg/ml LDL-treated cells. Results were analyzed by one- or two-way ANOVA and Tukey’s multicomparison test as indicated in the figure legends.

## RESULTS

### **Regulation of COX-2 and mPGES-1 mRNA and Protein Expression by a Combination of TNFα and PGE**_**2**_** and Increasing Concentrations of Native LDL**

**Fig. 1:**
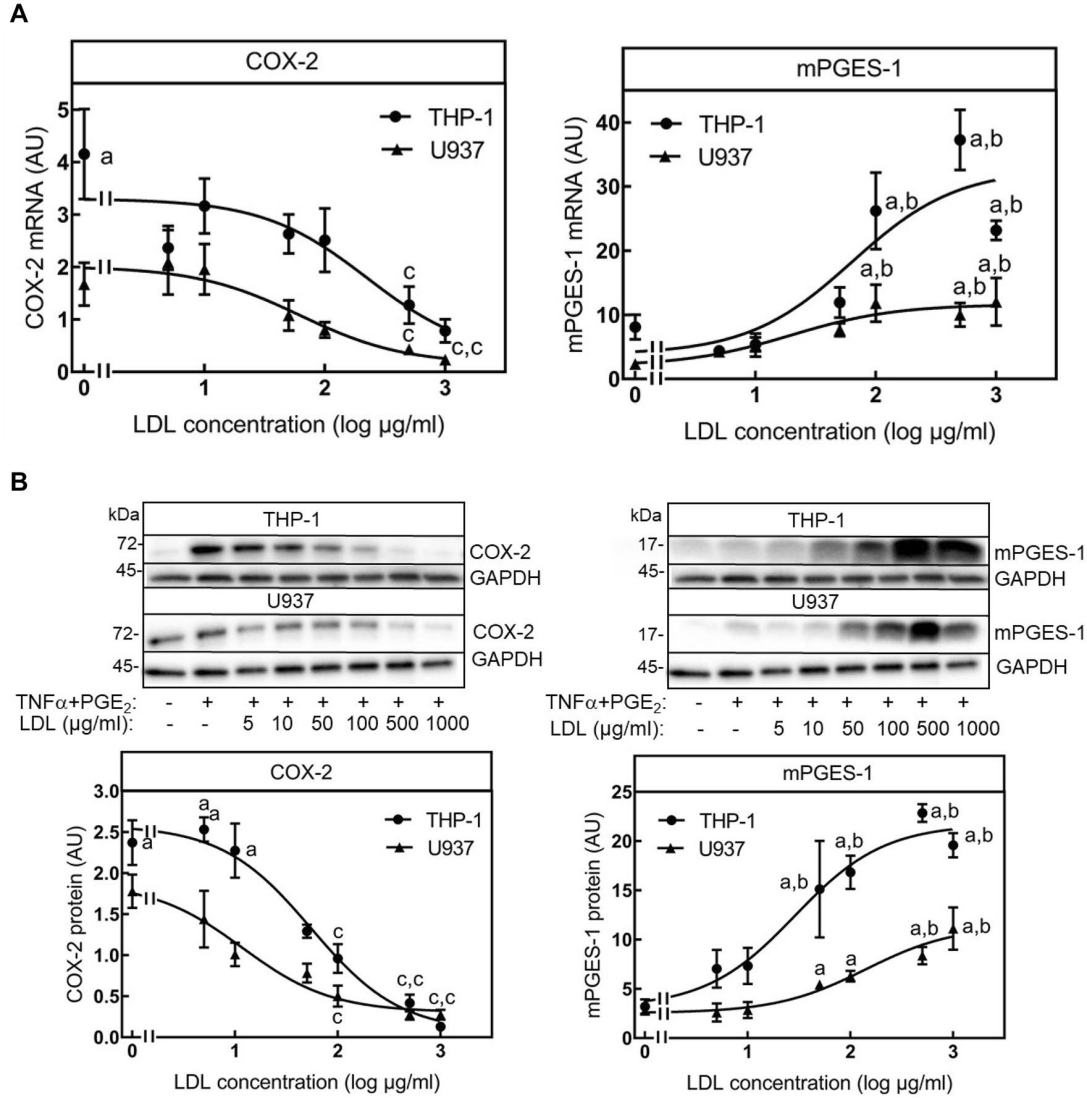
Dose-dependent modulation of TNFα/PGE_2_ induced COX-2 and mPGES-1 mRNA and protein expression by native LDL in THP-1 and U937 macrophages. THP-1 and U937 monocytes were differentiated to macrophages with 100 ng/ml PMA for 24 h and then incubated in culture medium containing 0.5% (v/v) FCS for another 24 h. Macrophages were then stimulated with 50 ng/ml TNFα and 1 µM PGE_2_ (TE) and increasing concentrations of native LDL for 24 h. **A** COX-2 and mPGES-1 mRNA content was measured by qPCR as described in the method section with GAPDH, actin, and HPRT as reference genes. **B** Proteins were extracted from cells with lysis buffer, and expression of COX-2 and mPGES-1 protein was measured by Western Blot using specific antibodies for COX-2 and mPGES-1, GAPDH as a housekeeping protein, peroxidase-coupled secondary antibodies, and a luminogenic substrate. Band intensity was quantified luminometrically. COX-2 and mPGES-1 protein was expressed as ratio between enzyme and GAPDH. Data shown are means ± SEM of at least five independent experiments performed in triplicate. Statistics: one-way ANOVA with Tukey’s multicomparison test: a, significantly higher than control; b, significantly higher than TE; c, significantly lower than TE (*p* < 0.05).

THP-1 and U937 monocytes were differentiated into macrophages, serum starved for 24 h, and cultured with a combination of TNFα and PGE_2_ with increasing concentrations of native LDL for 24 h. PGE_2_ and TNFα were used as a proinflammatory stimulus as both molecules are upregulated in inflammatory processes at the same time and in former studies the combination of TNFα and PGE_2_ led to maximal induction of the proinflammatory chemokine IL-8 in monocytes [21]. COX-2 and mPGES-1 mRNA expression was measured by real-time PCR (A) and protein expression by Western blot (B). The combination of TNFα and PGE_2_ induced COX-2 mRNA expression in THP-1 (4-fold) but only slightly in U937 macrophages (2-fold) (Fig. 1A). Both inductions were statistically not significant. Stimulation with native LDL repressed TNFα + PGE_2_-induced COX-2 mRNA expression slightly starting at 10 µg/ml and significantly at concentrations of 500–1000 µg/ml in both cell lines (Fig. 1A). mPGES-1 mRNA expression was induced by a combination of TNFα and PGE_2_ in both cell lines (THP-1: 8-fold; U937: 2-fold). Both inductions were not significant. Surprisingly, and in contrast to COX-2 mRNA expression, native LDL increased mPGES-1 mRNA expression in both cell lines significantly at concentrations greater than or equal to 100 µg/ml. The maximal induction was observed at 500 µg/ml (Fig. 1A).

Similar to the regulation of COX-2 mRNA, the combination of TNFα and PGE_2_ induced COX-2 protein slightly but not significant in THP-1 and U937 macrophages (2-fold) (Fig. 1B). In line with COX-2 mRNA native LDL dose-dependently decreased COX-2 protein expression both in THP-1 and U937 macrophages. The inhibition was significant with concentrations of 100 µg/ml and above. Maximal inhibition was achieved with 500 µg/ml native LDL. Similar to COX-2, the expression of mPGES-1 was slightly but not significantly induced by the combination of TNFα and PGE_2_ in THP-1 and U937 cells (3-fold). In line with the regulation of mPGES-1 mRNA native LDL dose-dependently enhanced mPGES-1 protein expression in both cell lines being maximal at 500 g/ml LDL (THP-1: 2010-fold and U937: 10-fold) (Fig. 1B). In summary, native LDL regulates COX-2 and mPGES-1 in an opposite manner in the two macrophage cell lines: Whereas COX-2 expression was repressed on both the mRNA and the protein levels, mPGES-1 expression was enhanced by native LDL.

### Modulation of Cell Viability and Induction of Apoptosis by Increasing LDL Concentrations

As repression of COX-2 and induction of mPGES-1 expression was achieved predominantly at higher LDL-concentrations, cell viability under these conditions was analyzed by cell-dependent resazurin to resorufin conversion (Alamar Blue assay). Induction of apoptosis was determined by capase-3 cleavage in lysates of treated cells. As shown in supplemental Fig. 1, LDL in combination with TNFα and PGE_2_ decreased resazurin to resorufin conversion in viable cells at 500 µg/ml and 1000 µg/ml by 20–30% in THP-1 and U937 macrophages. However, this reduction was statistically not significant. Stimulation of THP-1 macrophages with concentration ≥ 100 µg/ml induced caspase-3 cleavage slightly but not significantly (Supplemental Fig. 2). No cleavage was observed in U937 cells. In conclusion, LDL affected COX-2 and mPGES-1 expression already at concentrations that caused no significant reduction of cell viability.

**Fig. 2:**
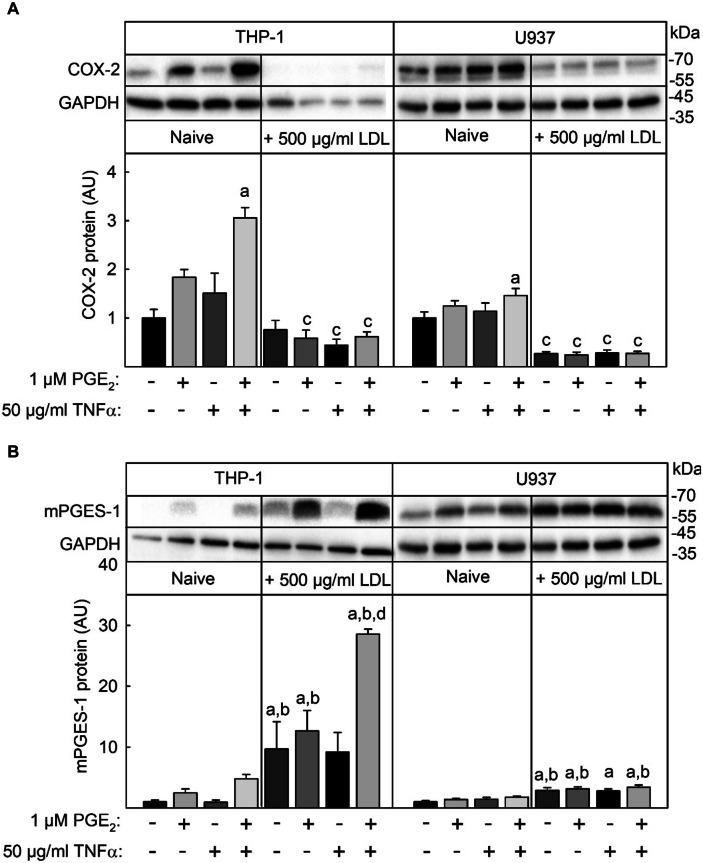
Modulation of TNFα, PGE_2_, or TNFα and PGE_2_ induced COX-2 and mPGES-1 mRNA and protein expression by native LDL in THP-1 and U937 macrophages. THP-1 and U937 monocytes were differentiated and cultured described in the legend of Fig. 1. Macrophages were then stimulated with 50 ng/ml TNFα, 1 µM PGE_2_, or 50 ng/ml TNFα + 1 µM PGE_2_ in the absence or presence of 500 µg/ml native LDL for 24 h. COX-2 (**A**) and mPGES-1 (**B**) protein content was measured as described in the legend of Fig. 1B. Data shown are means ± SEM of at least five independent experiments performed in triplicate. Statistics: two-way ANOVA with Tukey’s multicomparison test: a, significantly higher than control; b, significantly higher than without 500 µg/ml LDL; c, significantly lower than without 500 µg/ml LDL; d, significantly higher than TNFα + 500 µg/ml LDL or PGE_2_ + 500 µg/ml LDL (*p* < 0.05).

### **Interaction of Native LDL, TNFα, and PGE**_**2**_** in the Regulation of COX-2 and mPGES-1 Expression**

To analyze the interaction of native LDL, TNFα, and PGE_2_ in the regulation of COX-2 and mPGES-1 expression in more detail, THP-1 and U937 macrophages were stimulated wit 50 µg/ml TNFα and 1 µM PGE_2_ alone or in combination in the absence or presence of 500 µg/ml native LDL. Then, COX-2 and mPGES-1 expression was analyzed on the protein level. In THP-1 and U937 macrophages, only the combination of TNFα and PGE_2_ significantly induced COX-2 expression whereas mPGES-1 expression by TNFα and PGE_2_ was not significant in both cell lines (Fig. 2A, B). Induction of COX-2 protein expression was repressed by 500 µg/ml native LDL in both cell lines. Native LDL alone induced mPGES-1 protein expression significantly in both cell lines (THP-1: 10-fold, U937: 3-fold). This induction was enhanced only by the combination of TNFα and PGE_2_ in THP-1 macrophages but not in U937 cells.

In summary, the induction of both enzymes which are important in the production of PGE_2_ was maximal by the simultaneous stimulation with TNFα and PGE_2_. In THP-1 but not U937 cells, induction of COX-2 and mPGES-1 by PGE_2_ seems to be more important than by TNFα. Native LDL modulates this TNFα + PGE_2_-mediated enzyme induction in an opposite way: LDL repressed basal and TNFα + PGE_2_ induced expression of COX-2 and enhanced basal and induced expression of mPGES-1.

### THP-1 and U937 Macrophages Predominantly Expressed EP2 and EP4 Subtype

PGE_2_ acts upon a family of four different G-protein-coupled receptors called EP1 to EP4. To analyze which EP-R subtype might be involved in the induction of the LDL-modulated COX-2 and mPGES-1 induction by PGE_2_ in the macrophage cell lines, EP-R mRNA in these cells was quantified by real-time RT-PCR. To estimate exact EP-R mRNA copies, standard curves with plasmids containing defined copies of EP-R or GAPDH cDNA were prepared. EP2 mRNA was most abundant in both cell lines and was twice the expression of EP4 (Table 2). EP1 and EP3 mRNAs expression levels were very low in both macrophage cell lines. All EP mRNAs were expressed in THP-1 and U937 macrophages to a comparable level. It was, therefore, most likely that the PGE_2_-dependent induction of COX-2 and mPGES-1 expression was mediated via EP2 and EP4 receptors.

**Table 2:** EP-R mRNA Profile in THP-1 and U937 Macrophages

Cells	EP1	EP2	EP3	EP4
THP-1	0.03 ± 0.03	144.91 ± 66.97	5.62 ± 2.1	68.29 ± 17.36
U937	0.001 ± 0.0001	121 ± 35.95	2.49 ± 0.63	54.63 ± 14.35

THP-1 and U937 cells were differentiated and cultured as described in the legend to Fig. 1. EP-R mRNA and GAPDH mRNA of control cells was measured by real-time RT-qPCR as described in the “MATERIALS AND METHODS” section. Plasmids (10^2^–10^8^ copies) containing EP-R or GAPDH cDNAs were used for preparing standard curves for the calculation of EP-R or GAPDH mRNA copy numbers. Data represent the mean ± SEM of at least five independent RNA preparations. EP-R mRNA contents are expressed as copy number EP-R mRNA × 1000/copy number GAPDH mRNA.

### **Interaction of EP2/EP4 Agonists, TNFα**,** and Native LDL, in the Regulation of COX-2 and mPGES-1 Expression and **PGE_2_-synthesis** in THP-1 Macrophages**

**Fig. 3:**
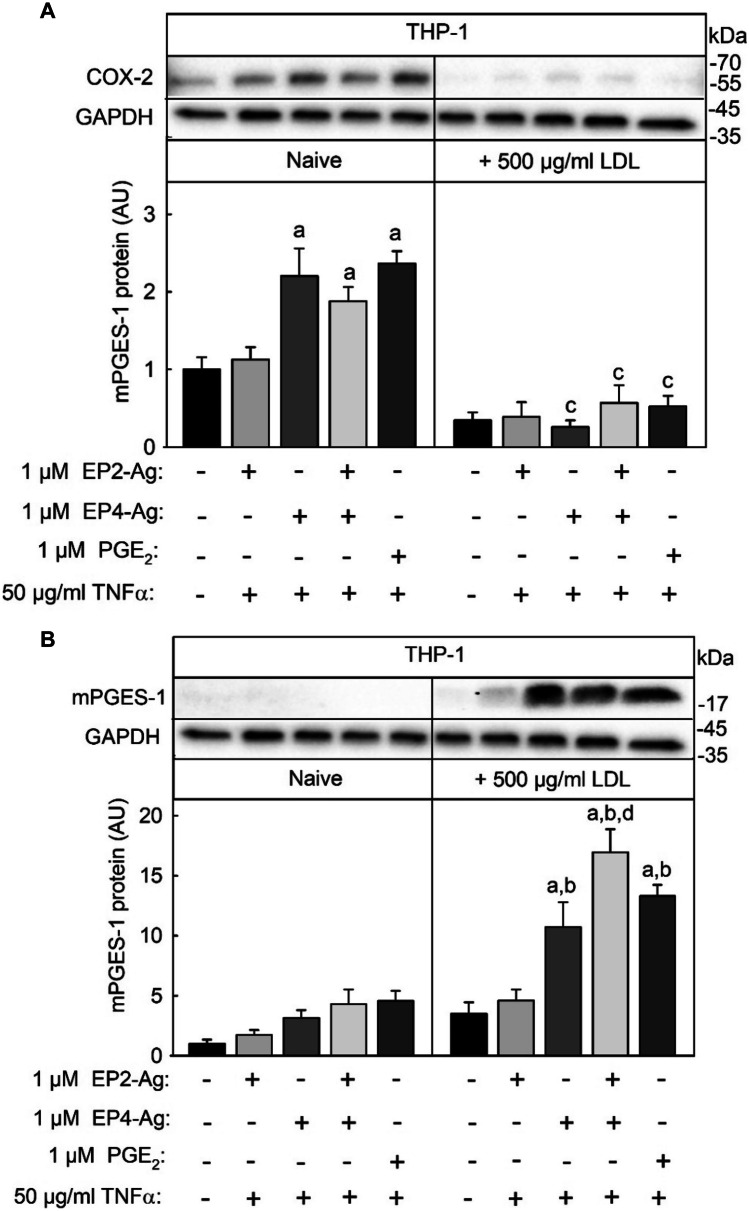
Modulation of EP2/EP-4 agonist, PGE_2_, and TNFα induced COX-2 and mPGES-1 protein expression by native LDL in THP-1 macrophages. THP-1 monocytes were differentiated and cultured described in the legend of Fig. 1. THP-1 macrophages were then stimulated with either 1 µM EP-2 or 1 µM EP-4 agonist alone or in combination + 50 ng/ml TNFα or 1 µM PGE_2_ + 50 ng/ml TNFα in the absence or presence of 500 µg/ml native LDL for 24 h. COX-2 (**A**) and mPGES-1 protein (**B**) was measured as described in the legend of Fig. 2. Data shown are means ± SEM of at least five independent experiments performed in triplicate. Statistics: two-way ANOVA with Tukey’s multicomparison test: a, significantly higher than control; b, significantly higher than without 500 µg/ml LDL; c, significantly lower than without 500 µg/ml LDL; d, significantly higher than EP4-agonist + TNFα + 500 µg/ml LDL (*p* < 0.05).

Since the regulation of COX-2 and mPGES-1 protein expression by TNFα and PGE_2_ and native LDL, as well EP-R expression was similar in THP-1 and U937 macrophages, all further experiments were performed solely on THP-1 cells. As the expression of EP1 and EP3 was very low in THP-1 cells, it was analyzed whether EP2 and or EP4 receptor agonists can mimic the effect of PGE_2_ in the regulation of COX-2 and mPGES-1 expression. THP-1 macrophages were stimulated with 50 µg/ml TNFα and either 1 µM of EP2 and EP4-agonist alone or in combination or 1 µM PGE_2_ and 50 ng/ml TNFα in the absence or presence of 500 µg/ml native LDL for 24 h. Then, COX-2 and mPGES-1 protein expression was analyzed. COX-2 expression was significantly induced to a similar level by TNFα in combination with the EP4-agonist alone, the combination of EP2/EP4-agonists and PGE_2_ (2-fold), but not in combination with the EP2-agonist alone (Fig. 3A). COX-2 expression, induced by the different combinations of stimuli, was significantly repressed by native LDL. The expression of mPGES-1 was also induced by TNFα in combination with the EP4-agonist alone, the combination of EP2/EP4-agonists and PGE_2_ (twofold) but to a lower level in combination with the EP2-agonist alone (Fig. 3B). However, these inductions were not significant. In combination with native LDL, TNFα and the EP4- but not the EP2-agonist significantly induced mPGES-1 expression to a comparable level as the stimulation with TNFα and PGE_2_. However, maximal mPGES-1 expression was induced with the combination of native LDL, TNFα, and both EP2/EP4-agonist.

The results show that COX-2 and mPGES-1 expression were induced in THP-1 cells by PGE_2_ mainly via EP4 receptor signal chains and that LDL can repress the signal chains leading to COX-2 expression and enhance the signal chains leading to mPGES-1 expression.

**Fig. 4:**
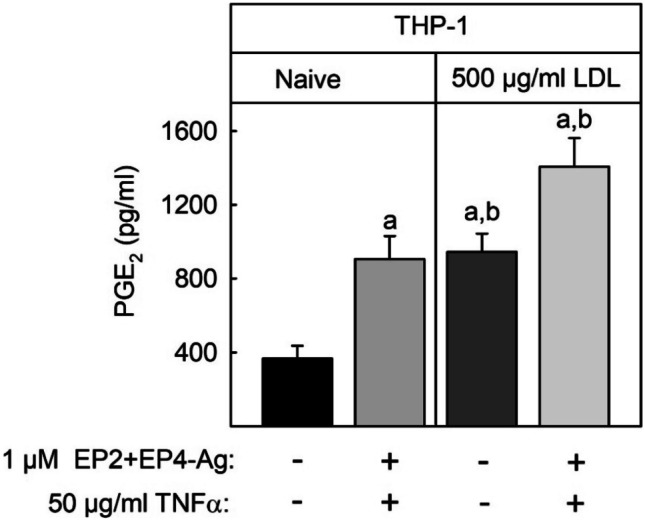
Modulation of PGE_2_ synthesis by EP-2/4 agonist, TNFα, and native LDL in THP-1 macrophages. THP-1 monocytes were differentiated, cultured, and stimulated as described in the legend of Fig. 3. PGE_2_ concentration in the supernatant of the cells was measured by competitive sandwich ELISA. Data shown are means ± SEM of at least five independent experiments performed in triplicate. Statistics: two-way ANOVA with Tukey’s multicomparison test: a, significantly higher than control; b, significantly higher than without 500 µg/ml LDL (*p* < 0.05).

Next, it was analyzed how PGE_2_-synthesis was regulated by EP2/EP4-agonists, TNFα, and native LDL. As observed for mPGES-1 mRNA and protein expression stimulation of THP-1 macrophages with EP2/EP4-agonists and TNFα as well as 500 µg/ml native LDL induced PGE_2_-synthesis to a comparable level (3-fold, Fig. 4). PGE_2_-synthesis was highest after simultaneous stimulation with EP2/4-agonist and TNFα and native LDL (6-fold). Therefore, PGE_2_-synthesis showed the same regulation pattern as mPGES-1 expression.

### Inhibition of LDL-Mediated Induction of mPGES-1 Expression by the PI3K Inhibitor LY294004

**Fig. 5:**
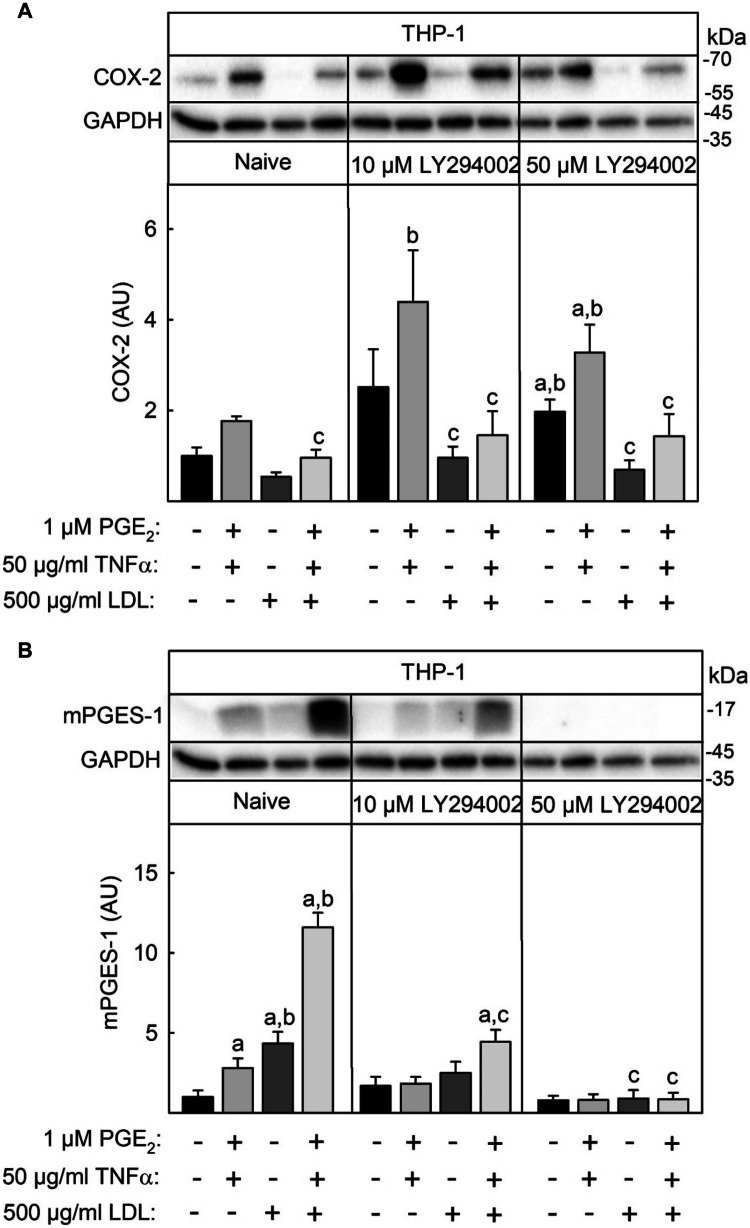
Inhibition of LDL-induced mPGES-1 expression by the PI3K inhibitor LY294002 in THP-1 macrophages. THP-1 macrophages were treated with 10 µM or 50 µM of the PI3K inhibitor Y294002 30 min before and during they were stimulated with 50 ng/ml TNFα + 1 µM PGE_2_ in the presence or absence of 500 µg/ml native LDL for 24 h. COX-2 (**A**) and mPGES-1 (**B**) were determined by Western blot as described in the legend of Fig. 2. Data shown are means ± SEM of at least five independent experiments performed in triplicate. Statistics: two-way ANOVA with Tukey’s multicomparison test: a, significantly higher than unstimulated control cells; b, significant higher than without LDL; c, significantly lower than naive, *p* < 0.05.

Expression of COX-2 and mPGES-1, which are the key enzymes in inflammation-induced PGE_2_ synthesis, is usually induced in concert by proinflammatory stimuli such as LPS and IL-1β by activation of transcription factor NFκB [22, 23]. An uncoupled expression of the two enzymes was described in primary rat microglia cells. In these cells, the PI3K inhibitors LY294002 or NVP-BEZ235 inhibited LPS-induced mPGES-1 expression and enhanced LPS-induced COX-2 expression [24, 25]. To analyze whether PI3K is also involved in the LDL-mediated repression of COX-2 vs. induction of mPGES-1, THP-1 macrophages were incubated with 10 µM or 50 µM LY294002 30 min before and during the stimulation with the combination of 50 ng/ml TNFα and 1 µM PGE_2_ in the absence or presence of 500 µM native LDL. Then, COX-2 and mPGES-1 expression was determined by Western blot. Preincubation with 10 µM LY294002 significantly reduced induction of mPGES-1 expression by the combination of TNFα and PGE_2_ in the presence of LDL (Fig. 5B). By contrast, LY294002 had no effect on the LDL-induced repression of COX-2 expression (Fig. 5A). In conclusion, PI3K may be involved in the LDL-mediated induction of mPGES-1 but not in the repression of COX-2.

### LDL-Mediated Activation of Akt-Kinase in THP-1 Macrophages

**Fig. 6:**
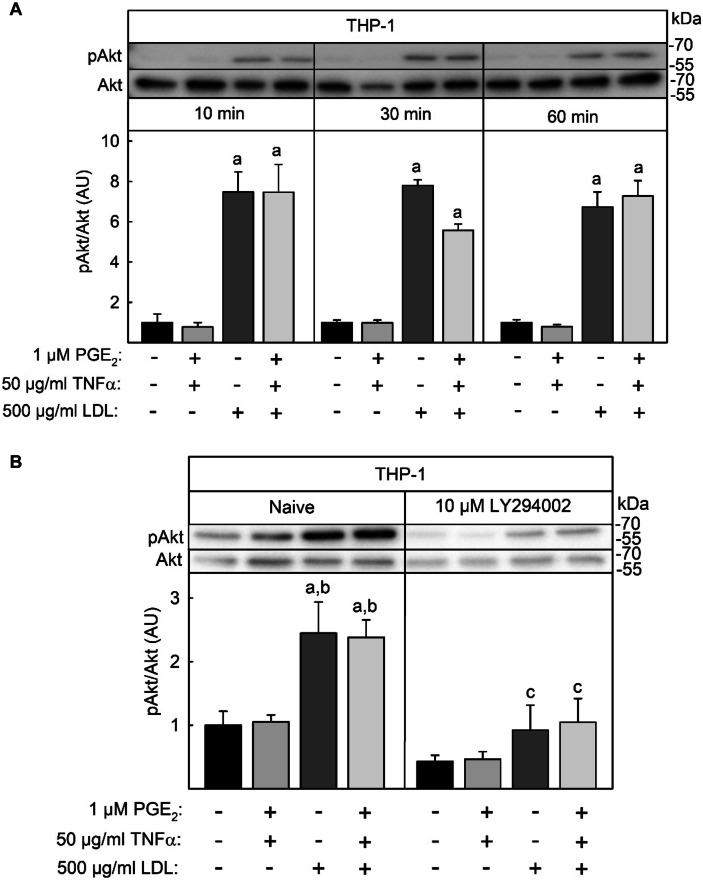
LDL-induced Akt kinase phosphorylation in THP-1 macrophages. **A** THP-1 macrophages were stimulated with 50 ng/ml TNFα + 1 µM PGE_2_ in the presence or absence of 500 µg/ml native LDL for the times indicated. **B** THP-1 macrophages were treated the PI3K inhibitor Y294002 30 min before and during they were stimulated with 50 ng/ml TNFα + 1 µM PGE_2_ in the presence or absence of 500 µg/ml native LDL for 24 h as described in the legend of Fig. 5. Proteins were extracted from cells with lysis buffer containing fluoride and vanadate to inhibit phosphatases. Phosphorylated and total Akt kinase were determined by Western blot using specific antibodies, peroxidase-coupled secondary antibodies, and a luminogenic substrate. Band intensity was quantified luminometrically and expressed as ratio between phosphorylated and total protein. Data shown are means ± SEM of at least five independent experiments performed in triplicate. Statistics: two-way ANOVA with Tukey’s multicomparison test: a, significantly higher than unstimulated control cells; b, significantly higher than without LDL; c, significantly lower than naive, *p* < 0.05.

As inhibition of PI3K repressed LDL-mediated induction of mPGES-1 in THP-1 macrophages, this point to a direct activation of PI3K by native LDL. A down-stream target substrate of activated PI3K is the Akt-kinase, which is activated by PI3K-mediated phosphorylation. Therefore, LDL-mediated Akt phosphorylation was analyzed by Western blot using a phospho-specific Akt antibody. Stimulation of THP-1 macrophages with 500 µg/ml native LDL for 10 min led to significant Akt-phosphorylation which was constant at 30 and 60 min and was not effected by simultaneous stimulation with TNFα and 1 µM PGE_2_ (Fig. 6A). In addition, a significant LDL-mediated Akt phosphorylation was also measured at the end of the 24 h LDL stimulation period and was suppressed by the PI3K inhibitor LY294002 (Fig. 6B). In conclusion, LDL-mediated induction of mPGES-1 expression is most likely a result of LDL-mediated PI3K activation.

### LDL-Mediated Inhibition of Egr-1 Expression and ERK-Phosphorylation

**Fig. 7:**
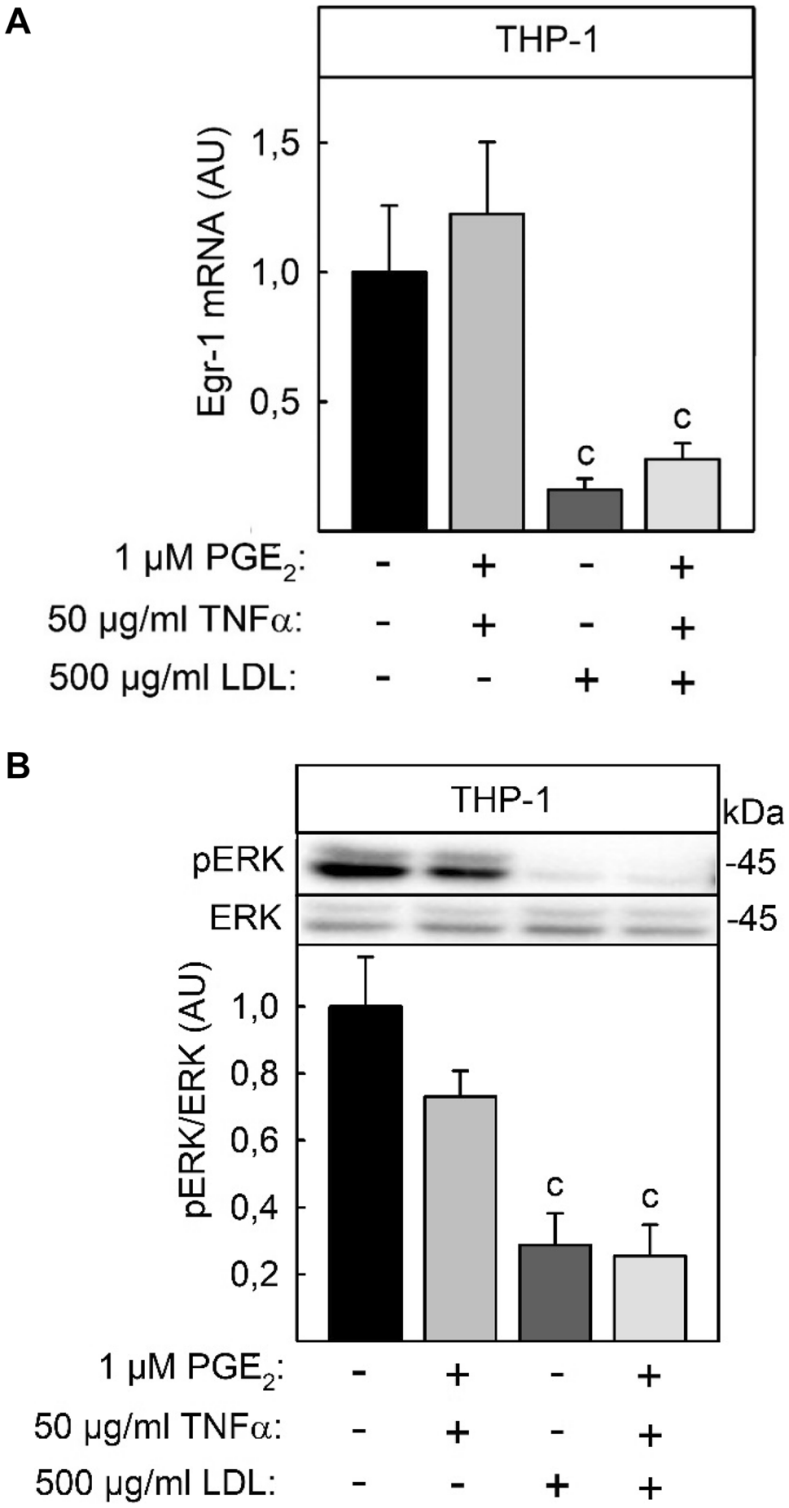
LDL-repressed Egr-1 expression and reduction of ERK kinase phosphorylation in THP-1 macrophages. THP-1 macrophages were stimulated with 50 ng/ml TNFα + 1 µM PGE_2_ in the presence or absence of 500 µg/ml native LDL for the times indicated. **A** Phosphorylated and total ERK kinase were determined by Western blot as described in the legend of Fig. 7. **B** Egr-1 mRNA content was measured as described in the legend of Fig. 1. Data shown are means ± SEM of at least five independent experiments performed in triplicate. Statistics: two-way ANOVA with Tukey’s multicomparison test: c, significant lower than without LDL, *p* < 0.05.

In contrast to rat microglia cells, where PI3K inhibitors simultaneously induced COX-2 and repressed mPGES-1 expression, LY294002 did not reverse LDL-mediated repression of COX-2 expression. So, there is some evidence that native LDL repressed COX-2 through other signal chains. An important transcription factor which positively controls both COX-2 and mPGES-1 expression is the transcription factor early growth response protein 1 (Egr-1) [23, 26]. Egr-1 is induced by proinflammatory stimuli such as LPS or IL-1β [23, 27]. So, next it was examined whether native LDL can modulate Egr-1 expression. Egr-1 mRNA expression was not modulated by stimulation with the combination of TNFα and PGE_2_. By contrast, 500 µg/ml native LDL significantly repressed Egr-1 mRNA expression both in control and TNFα and 1 µM PGE_2_ stimulated THP-1 macrophages (Fig. 7A). Besides the induction of Egr-1 expression by proinflammatory stimuli, Egr-1 expression is also controlled by extracellular-signal regulated kinase ERK which is activated by phosphorylation and deactivated through dephosphorylation. Through Western blotting with a phospho-secific ERK antibody, it was determined that native LDL can modulate the phosphorylation status of ERK. Similar to Egr-1 mRNA expression, the basal phosphorylation of ERK, which was uninspected high in PMA-treated, serum-starved THP-1 cells, was not influenced by stimulation with the combination of TNFα and PGE_2_ but was significantly repressed by stimulation with 500 µg/ml native LDL for 24 h (Fig. 7B). Therefore, it was assumed that LDL-mediated repression of COX-2 was a result of LDL-mediated repression of ERK phosphorylation and Egr-1 expression.

### LDL-Mediated Modulation of NFκB Signalling

Coordinated expression of COX-2 and mPGES-1 by LPS and IL-1β is mainly controlled by transcription factor NFκB [23]. One of the important steps in NFκB activation is the activation of IKK by phosphorylation. Then, phosphorylated IKK can phosphorylate IκB which leads to its ubiquitinylation and proteasomal degradation and NFκB activation. Therefore, we measured IKK phosphorylation by TNFα + PGE_2_ and increasing concentrations of LDL in lysates of THP-1 macrophages by western blot. As shown in Supplemental Fig. 3 treatment with TNα + PGE_2_ increased the amount of pIKK slightly but not significantly. IKK phosphorylation was marginally and also not significantly repressed by increasing LDL concentrations. So, activation of NFκB signal chain seems not to be involved in the LDL-mediated regulation of COX-2 and mPGES-1 expression.

## DISCUSSION

This current study showed that simultaneous activation of TNFα-R and EP2/EP4 signal chains induced COX-2 and mPGES-1 mRNA and protein expression in the macrophage cell lines THP-1 and U937. Surprisingly, native LDL repressed COX-2 expression and markedly enhanced mPGES-1 as well as PGE_2_ synthesis. In THP-1 cells, LDL-mediated induction of mPGES-1 expression was most likely induced by PI3K activation, whereas COX-2 repression may be a functional consequence of reduced ERK phosphorylation and expression of transcription factor Egr-1.

### Role of Native LDL in the Inflammatory Response

A hallmark of the development of atherosclerosis is the uptake of modified LDL, which undergoes spontaneous oxidation or modification, by macrophages via scavenger receptors such as CD36 or SR-A, resulting in the formation of foam cells. Beside foam cell formation, oxidized LDL also triggers a chronic inflammatory response, which is a confirmed main cause of plaque rupture and thrombosis [28]. Macrophages in atherosclerotic lesions are key players in the inflammatory process as they produce proinflammatory cytokines and eicosanoids [29]. Only little is known about the function of native LDL in atherosclerotic inflammatory response. Different from oxidized LDL, native LDL is bound and internalized by the LDL-R. Its expression is under tight control of the intracellular cholesterol concentration: an increase in cellular cholesterol downregulates LDL-R expression via a negative feedback loop [30].

Interestingly, most studies that analyzed the influence of native LDL on the inflammatory response in macrophages showed that native LDL rather inhibited than enhanced inflammation. In THP-1 macrophages, native LDL dose-dependently inhibited the serum amyloid A (SAA), induced IL-1β and TNFα expression, most likely by inhibiting NLRP3 inflammosome activation by SAA [31]. A mixture of native LDL, VLDL, and HDL (VLR) also inhibited the basal expression of several inflammatory genes such as IL-6, IL1β, and IL-23A [32]. By contrast to the inhibition of SAA-induced inflammation, the authors of this study postulated that native LDL inhibited expression of inflammatory genes by hypermethylation of their promotor region, whereas LDL had no effect on histone acetylation. In line with these two reports, degradation of the LDL-R in THP-1 macrophages by PCSK9 led to a proinflammatory response, inducing the expression of the proinflammatory cytokines IL-1β, TNFα, MCP-1, and CXCL2 [33].

The aim of the current study was to analyze whether native LDL can modulate the expression of COX-2 and mPGES-1. However, although the experiments were performed with purified, native LDL, spontaneous oxidation during the experiment cannot be entirely excluded. Thus, we determined the oxidation status of the native LDL using malondialdehyde (MDA) assay as an indicator of lipid peroxidation (Supplemental Fig. 4). Whereas MDA was not detectable in the culture medium, the addition of 500 µg/ml LDL, which was not actively modified by chemical oxidation, significantly increased MDA concentration. MDA concentration was markedly enhanced by 24-h incubation with THP-1 macrophages (5-fold). It can, therefore, not be excluded that oxidized LDL also contributed to the regulation of COX-2 and mPGES-1.

The effect of mPGES-1 expression on the development of inflammatory disease as atherosclerosis was analyzed using mPGES-1 knockout mice. Global deletion of mPGES-1 in an LDL-R (-/-), fat-fed model slows atherogenesis, which is indicated by a reduced plaque burden and a reduced number of macrophage foam cells in atherosclerotic lesions [34]. Cell-specific deletion of the mPGES-1 revealed that PGE_2_ formation in macrophages appears to be essential in this process: Mice lacking mPGES-1 expression in myeloid cells showed markedly reduced atherogenesis in an LDL-R (-/-), high fat-fed model, whereas depletion of mPGES-1 in vascular-smooth muscle or endothelial cells did not alter development of atherosclerosis in this mouse model [35]. These results implicate, that the LDL-mediated induction of mPGES-1 expression (Fig. 1) and enhanced PGE_2_-secretion (Fig. 4) observed in the current study might increase development of atherosclerosis, especially in combination with other inflammatory mediators such as TNFα and PGE_2_ which induced maximal expression of the chemotactic cytokine IL-8 in monocytic cell lines and PBMC [21].

### **Increase of **PGE_2_-synthesis ** After LDL-Mediated Concomitant mPGES-1 Induction and COX-2 Repression**

For an effective synthesis of PGE_2_, in most cases, the expression of mPGES-1, which generate PGE_2_ from PGH_2_, is functionally coupled to expression of COX-2, which provide PGH_2_ by conversion of arachidonic acid. Coordinated induction of expression of COX-2 and mPGES-1 by pro-inflammatory stimuli such as IL-1β, TNFα, or LPS, activating transcription factor NFκB or PGE_2_, via activation of transcription factor CREB was observed in macrophages [36]. In the current study, native LDL markedly decreased COX-2 but enhanced mPGES-1 expression, both induced by the combination of TNFα and PGE_2_. Native LDL stimulated PGE_2_ synthesis and enhanced PGE_2_-synthesis induced by the combination of TNFα andEP2/EP4 agonists. Since LDL repressed COX-2 the question arose, which pathway might furnish PGH_2_ as substrate for the subsequent mPGES-1 reaction? One possible explanation might be the high constitutive expression of COX-1 in THP-1 cells (Table 3). In the current study, the COX-1 mRNA copy numbers in naive or TNFα and PGE_2_ stimulated THP-1 macrophages were comparable to COX-2 mRNA (Table 3). A robust COX-1 expression in THP-1 macrophages was also confirmed in other publications [37, 38]. Therefore, COX-1 rather than COX-2 may generate PGH_2_ in THP-1 macrophages, which was converted to PGE_2_ by LDL-induced mPGES-1.

**Table 3:** COX mRNA Profile in THP-1 Macrophages

COX-1 Control	COX-1 TNFα + PGE_2_	COX-2 Control	COX-2 TNFα + PGE_2_
3.14 ± 1.27	6.09 ± 1.97	2.48 ± 1.57	11.9 ± 4.29

THP-1 cells were differentiated and cultured as described in the legend to Fig. 1. COX mRNA and GAPDH mRNA of control and TNFα + PGE_2_-stimulated cells was measured by real-time RT-qPCR as described in the “MATERIALS AND METHODS” section. Plasmids (10^2^–10^8^ copies) containing COX or GAPDH cDNAs were used for preparing standard curves for the calculation of COX or GAPDH mRNA copy numbers. Data represent the mean ± SEM of at least five independent RNA preparations. COX mRNA contents are expressed as copy number COX/PGES mRNA × 1000/copy number GAPDH mRNA.

### LDL-Stimulated Signal Chains Leading to Induction of mPGES-1 Expression and Repression of COX-2 Expression

In the current study, native LDL repressed the induction of TNFα + PGE_2_-induced COX-2 expression and simultaneously enhanced the expression of mPGES-1 (Fig. 1). Most studies analyzing the regulation of COX-2 and mPGES-1 expression show a coordinated expression of both enzymes. Expression of both enzymes in macrophages were induced by LPS or IL-1β mediated activation of transcription NFκB as well as PGE_2_-mediated CREB activation [23, 36]. A simultaneous induction of COX-2 and mPGES-1 was also found by activation of the NLRP3 inflammasome [39] and by LPS [40]. By contrast, COX-2 and mPGES-1 expression in rat glia cells were regulated in an uncoupled way using inhibitors of PI3K. Whereas LPS-induced COX-2 expression was enhanced by stimulation of PI3K, LPS-induced expression of mPGES-1 was repressed, leading to an overall reduction of PGE_2_ and an induction of PGD_2_-release [24, 25]. To prove whether LDL-mediated repression of COX-2 and enhanced expression of mPGS-1 was due to an activation of PI3K, activation of the kinase by LDL was analyzed measuring phosphorylation of the PI3K substrate Akt kinase (Fig. 6) as well as using a PI3K inhibitor (Fig. 5). In line with this hypothesis first, stimulation of THP-1 macrophages led to a phosphorylation of Akt kinase (Fig. 6) and secondly, the LDL-mediated enhancement of mPGES-1 expression was completely blocked in THP-1 cells using the pan PI3K inhibitor LY294002 (Fig. 5). These data strongly indicate, for the first time, that native LDL enhanced mPGES1 expression in activated macrophages by a PI3K-dependent signal chain. An LDL-mediated activation of Akt kinase was also described in human endothelial cells [41]. In these cells, native LDL activated a PI3K-Akt-mTOr signalling pathway most likely by the formation of an LDL-R/ insulin receptor complex. Activation of this signal chain led to an LDL-mediated, insulin-mimetic glucose uptake in the endothelial cells.

By contrast to mPGES-1 expression, the PI3K inhibitor did not influence LDL-mediated repression of COX-2 expression in activated THP-1 macrophages. One of the key factors in the induction of COX-2 expression is the transcription factor Egr-1, which is transcriptionally controlled by an activated ERK kinase [42, 43]. The current study indicated that native LDL repressed Egr-1 expression and abolished ERK phosphorylation in THP-1 macrophages (Fig. 7) which may explain the repression of COX-2 expression by LDL.

**Fig. 8:**
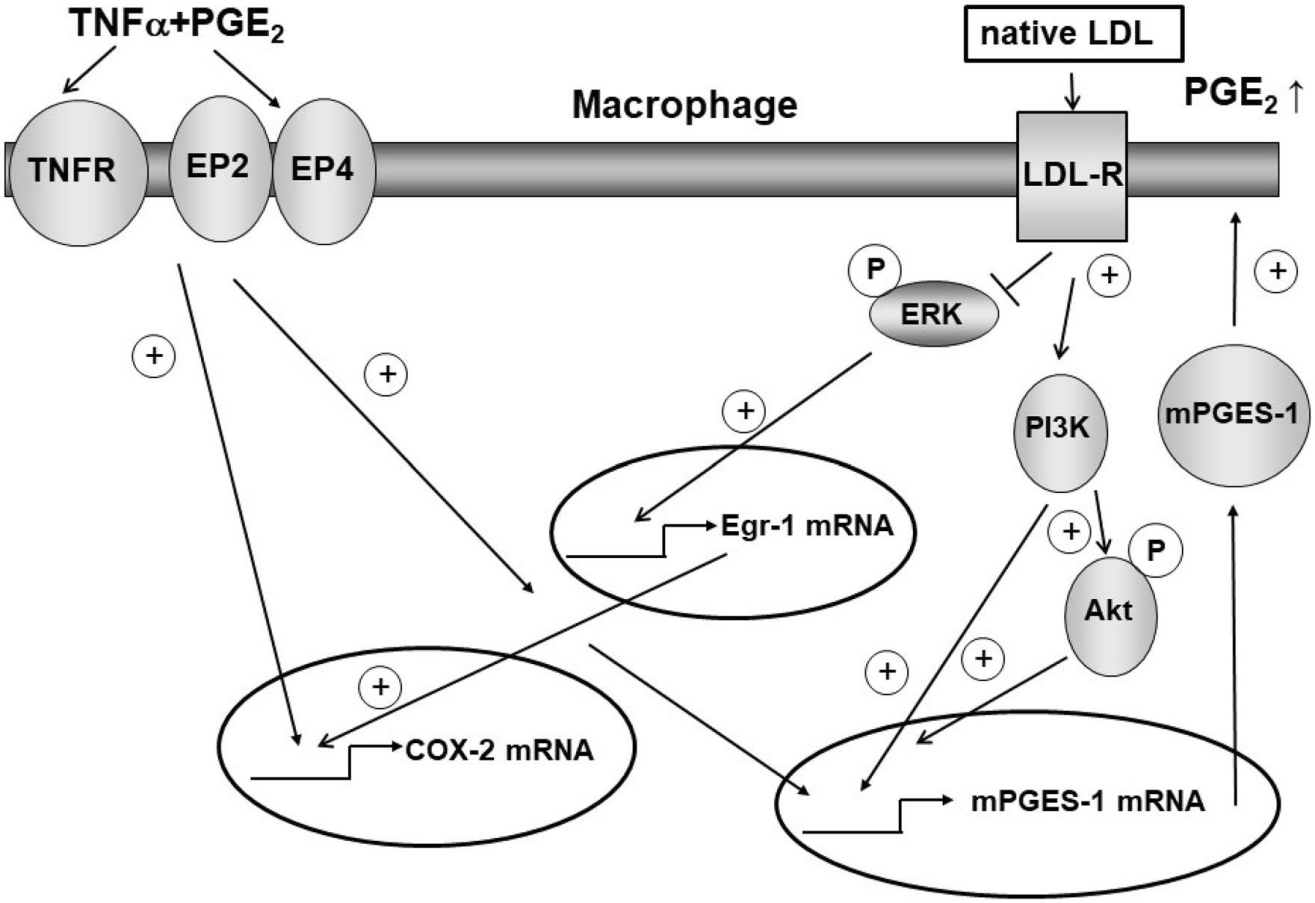
Model of the TNFα/PGE_2_/LDL-mediated regulation of COX-2 and mPGES1 expression in macrophage cell lines.

These findings suggest, for the first, time that in human macrophage cell lines LDL simultaneously repressed COX-2 and enhanced mPGES-1 expression induced by TNFα and PGE_2_ signal chains, most likely by inactivating pERK as well as activating PI3K (Fig. 8).

## Supplementary Information

Below is the link to the electronic supplementary material.

Supplementary file1 (DOCX 157 KB)

Supplementary file2 (DOCX 112 KB)

Supplementary file3 (DOCX 97.3 KB)

Supplementary file4 (DOCX 112 KB)

Supplementary file5 (DOCX 13 KB)

## Data Availability

All the data supporting the findings of the study were shown in this paper and are available upon reasonable request.

## Abbreviations

COX: Cyclooxygenase
GAPDH: Glycerinaldehyde 3-phosphate dehydrogenase
G protein: Guanine nucleotide-binding protein
EP1-4: Prostaglandin E_2_ receptor subtype 1–4
I-κB: Inhibitor of kappaB
IKK: Inhibitor of kappaB kinase
LPS: Lipopolysaccharid
mPGES-1: Microsomal prostaglandin E_2_ synthase 1
NF-κB: Nuclear factor-kappa B
PGE_2_: Prostaglandin E_2_
PKA: Protein kinase A
PKC: Protein kinase C
PI3K: Phosphoinositid 3 Kinase
TNFα: Tumor Necrosis Factor-α
TNFα-R: Tumor Necrosis Factor-α receptor
