# Supplementary material for: LDL-Dependent Regulation of TNFα/PGE2 Induced COX-2/mPGES-1 Expression in Human Macrophage Cell Lines
Source: Inflammation. 2023 Jan 4;46(3):893–911. doi: 10.1007/s10753-022-01778-y (PMC10188574; doi:10.1007/s10753-022-01778-y)
Supplement: Supplementary file 1 — Supplementary file1 (DOCX 157 KB) [file 10753_2022_1778_MOESM1_ESM.docx]

**Supplemental Fig 1:**


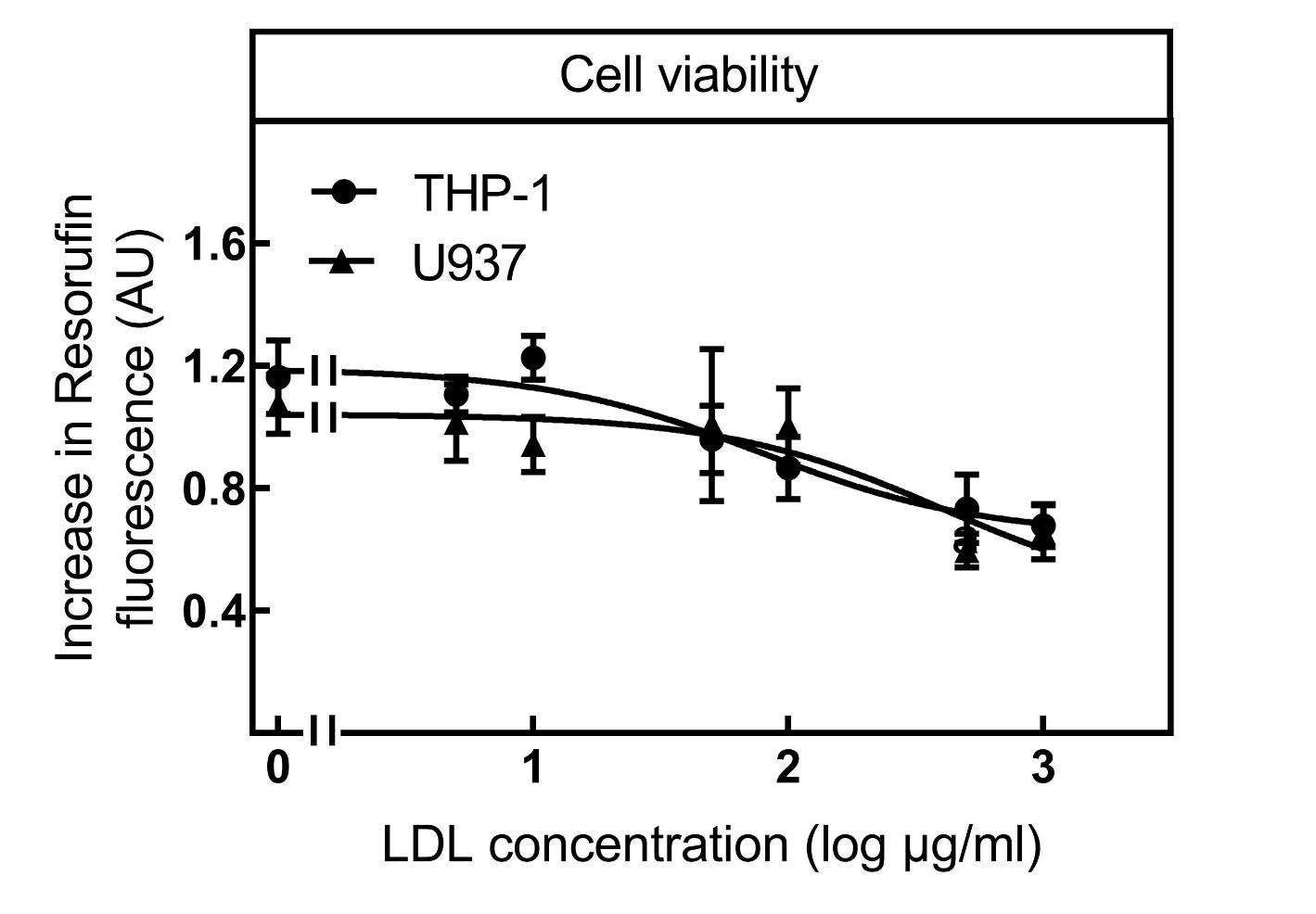


**Supplemental Fig. 1: Dose-dependent modulation of resazurin to resorufin conversion (Alamar Blue) by native LDL together with TNFα and PGE_2_ in THP-1 and U937 macrophages**. THP-1 and U937 monocytes were differentiated to macrophages with 100 ng/ml PMA for 24 h and then incubated in culture medium containing 0.5 % (v/v) FCS for another 24 h. Macrophages were then stimulated with 50 ng/ml TNFα and 1 µM PGE_2_ (TE) and increasing concentrations of native LDL for 24 h. Then cells were washed and incubated with 0,1 mg/ml resazurin in culture medium for 2 h. Resorufin fluorescence was measured every 30 min (Em 544 nm;Exc 590 nm). Data shown are means + S.E.M. of at least four independent experiments performed in triplicate. Statistics: 1-way ANOVA with Tuckey’s multicomparison test.
