# Supplementary material for: LDL-Dependent Regulation of TNFα/PGE2 Induced COX-2/mPGES-1 Expression in Human Macrophage Cell Lines
Source: Inflammation. 2023 Jan 4;46(3):893–911. doi: 10.1007/s10753-022-01778-y (PMC10188574; doi:10.1007/s10753-022-01778-y)
Supplement: Supplementary file 2 — Supplementary file2 (DOCX 112 KB) [file 10753_2022_1778_MOESM2_ESM.docx]

**Supplemental Fig 2:**


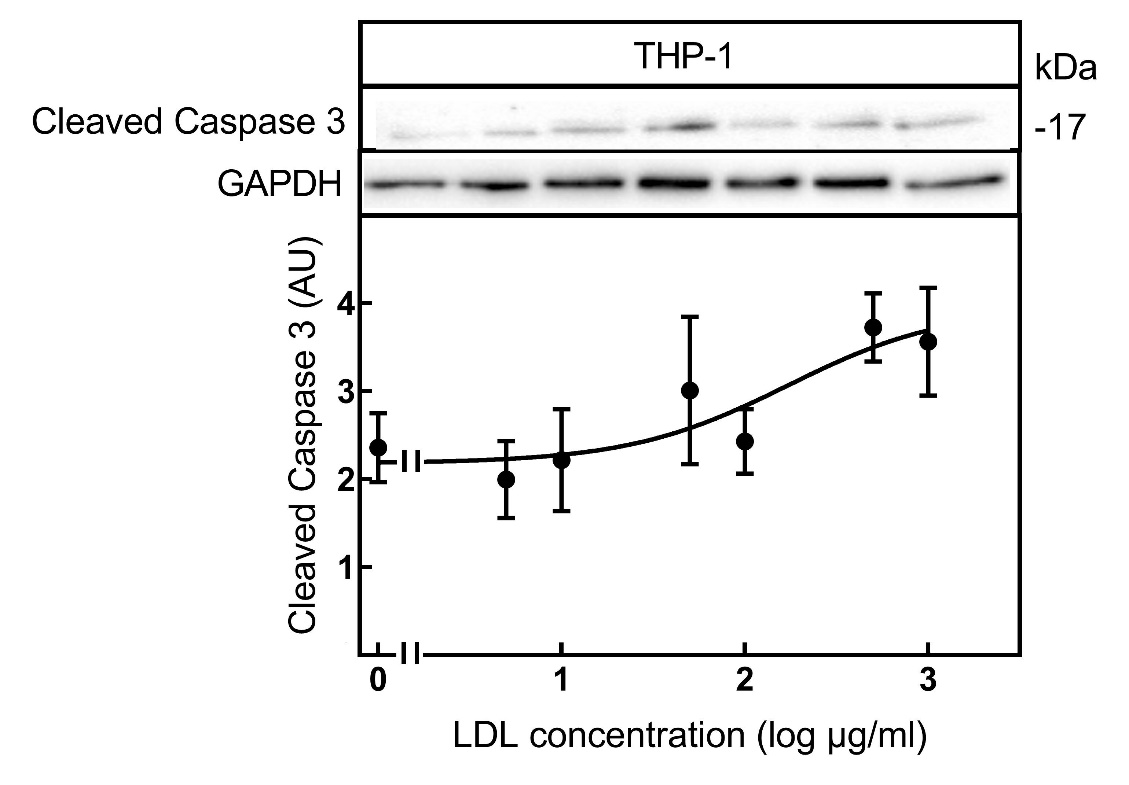


**Supplemental Fig. 2: Dose-dependent modulation of caspase 3 cleavage by native LDL together with TNFα and PGE_2_ in THP-1 macrophages**. THP-1 monocytes were differentiated to macrophages with 100 ng/ml PMA for 24 h and then incubated in culture medium containing 0.5 % (v/v) FCS for another 24 h. Macrophages were then stimulated with 50 ng/ml TNFα and 1 µM PGE_2_ (TE) and increasing concentrations of native LDL for 24 h. Then cells were washed and lysates were prepared as described in the legends of Fig. 1B. Caspase 3 cleavage, which indicates apoptosis, was determined using western blot with anti-cleaved caspase antibodies and GAPDH as reference protein. Data shown are means + S.E.M. of at least four independent experiments performed in triplicate. Statistics: 1-way ANOVA with Tuckey’s multicomparison test.
