# Supplementary material for: LDL-Dependent Regulation of TNFα/PGE2 Induced COX-2/mPGES-1 Expression in Human Macrophage Cell Lines
Source: Inflammation. 2023 Jan 4;46(3):893–911. doi: 10.1007/s10753-022-01778-y (PMC10188574; doi:10.1007/s10753-022-01778-y)
Supplement: Supplementary file 3 — Supplementary file3 (DOCX 97.3 KB) [file 10753_2022_1778_MOESM3_ESM.docx]

**Supplemental Fig 3:**


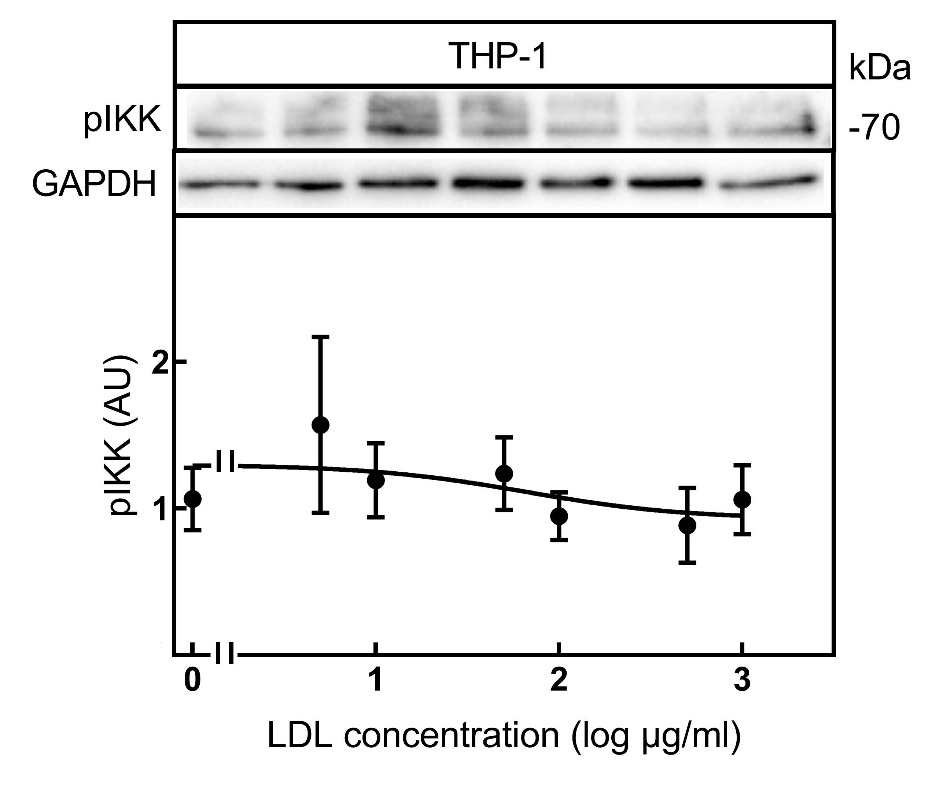


**Supplemental Fig. 3: Modulation of IKK phosphorylation by native LDL together with TNFα and PGE_2_ in THP-1 macrophages**. THP-1 monocytes were differentiated to macrophages with 100 ng/ml PMA for 24 h and then incubated in culture medium containing 0.5 % (v/v) FCS for another 24 h. Macrophages were then stimulated with 50 ng/ml TNFα and 1 µM PGE_2_ (TE) and increasing concentrations of native LDL for 24 h. Then cells were washed and lysates were prepared as described in the legends of Fig. 1B. IKK phosphorylation, which reflects activation of NFκB signalling pathway, was determined using western blot with anti-pIKK antibodies and GAPDH as reference protein. Data shown are means + S.E.M. of at least four independent experiments performed in triplicate. Statistics: 1-way ANOVA with Tuckey’s multicomparison test.
