# Supplementary material for: LDL-Dependent Regulation of TNFα/PGE2 Induced COX-2/mPGES-1 Expression in Human Macrophage Cell Lines
Source: Inflammation. 2023 Jan 4;46(3):893–911. doi: 10.1007/s10753-022-01778-y (PMC10188574; doi:10.1007/s10753-022-01778-y)
Supplement: Supplementary file 4 — Supplementary file4 (DOCX 112 KB) [file 10753_2022_1778_MOESM4_ESM.docx]

**Supplemental Fig 4:**


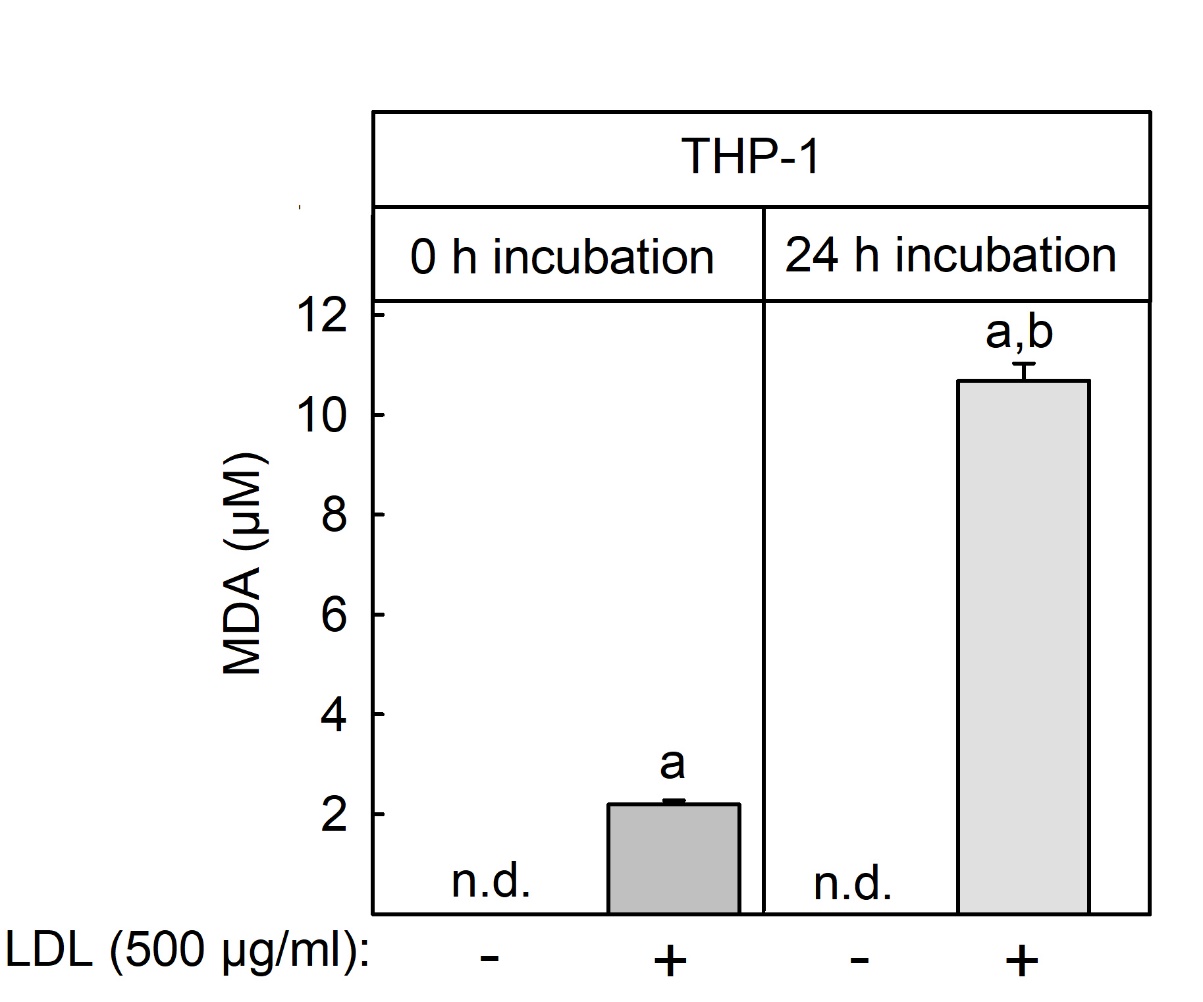


**Supplemental Fig. 4: Lipid peroxidation of LDL by THP-1 macrophages**. Differentiated THP-1 cells were incubated in culture medium (RPMI 16040 + 0,5% (v/v) FCS) with or without 500 µg/ml native LDL for 0 h or 24 h at 37°C. Malondialdehyde (MDA), as a product of lipid peroxidation, was measured in cell culture supernatants using a commercial TBARS assay kit (Cayman chemicals) according to the manufacturer’s instructions. Data shown are means + S.E.M. of three independent experiments performed in triplicate. Statistics: 2-way ANOVA with Tuckey’s multicomparison test. a: significant > than without LDL, b: significant > 0 h incubation (p < 0.05)
