# Supplementary material for: LDL-Dependent Regulation of TNFα/PGE2 Induced COX-2/mPGES-1 Expression in Human Macrophage Cell Lines
Source: Inflammation. 2023 Jan 4;46(3):893–911. doi: 10.1007/s10753-022-01778-y (PMC10188574; doi:10.1007/s10753-022-01778-y)
Supplement: Supplementary file 5 — Supplementary file5 (DOCX 13 KB) [file 10753_2022_1778_MOESM5_ESM.docx]

# Table S1 COX mRNA profile in THP-1 macrophages

| COX-1  Control | COX-1 TNFα+PGE_2_ | COX-2  Control | COX-2 TNFα+PGE_2_ |
| --- | --- | --- | --- |
| 3,14 + 1,27 | 6,09 + 1,97 | 2,48 + 1,57 | 11,9 + 4,29 |

THP-1 were differentiated and cultured as described in the legend to figure 1. COX mRNA and GAPDH mRNA of control and TNFα+PGE_2_-stimulated cells was measured by real-time RT-qPCR as described in *Methods*. Plasmids (102 – 108 copies) containing COX or GAPDH cDNAs were used for preparing standard curves for the calculation of COX or GAPDH mRNA copy numbers. Data represent the mean + SEM of at least five independent RNA preparations. COX mRNA contents are expressed as copy number COX/PGES mRNA x 1000/copy number GAPDH mRNA.
